# The Semiology of Motor Disorders in Autism Spectrum Disorders as Highlighted from a Standardized Neuro-Psychomotor Assessment

**DOI:** 10.3389/fpsyg.2016.01292

**Published:** 2016-09-12

**Authors:** Aude Paquet, Bertrand Olliac, Manuel-Pierre Bouvard, Bernard Golse, Laurence Vaivre-Douret

**Affiliations:** ^1^Faculty of Medicine, Paris Descartes University, Sorbonne Paris-CitéParis, France; ^2^Department of Child Psychiatry, AP-HP Necker-Enfants Malades University HospitalParis, France; ^3^Institut National de la Santé Et de la Recherche Médicale UMR 1018 and CESP, Universities of Paris-Saclay and Paris-Sud, UVSQVillejuif, France; ^4^Department of Child and the Adolescent Psychiatry, Esquirol HospitalLimoges, France; ^5^INSERM UMR 1094, Tropical Neuroepidemiology, University of LimogesLimoges, France; ^6^Department of Child and the Adolescent Psychiatry, Perrens Hospital, Université de BordeauxBordeaux, France; ^7^Centre National de la Recherche Scientifique UMR 5287, Institut de Neurosciences Cognitives et Intégratives d'Aquitaine, University of BordeauxBordeaux, France; ^8^Department of Pediatrics, Child development, Cochin-Port Royal University Hospitals, Assistance Publique-Hôpitaux de ParisParis, France; ^9^Endocrinology Laboratory, Imagine Institut, Necker-Enfants Malades University HospitalParis, France

**Keywords:** Autism Spectrum Disorders (ASD), children, motor disorder, neuro-psychomotor functions, neurodevelopmental assessment

## Abstract

**Background:** Altered motor performance has been described in Autism Spectrum Disorders (ASD) with disturbances in walking; posture, coordination, or arm movements, but some individuals with ASD show no impairment of motor skills. The neuro-developmental processes that underpin the performance of neuro-psychomotor functions have not been widely explored, nor is it clear whether there are neuro-psychomotor functions specifically affected in ASD. Our objective was to focus on the semiology of motor disorders among children with ASD using a neuro-developmental assessment tool.

**Method:** Thirty-four children with ASD, with or without intellectual deficit (ID) were recruited in a child psychiatry department and Autism Resource Centers. Initial standard evaluations for diagnosis (psychiatric; psychological; psychomotor) were supplemented by a standardized assessment battery for neuro-developmental psychomotor functions (NP-MOT).

**Results:** The results of some NP-MOT tests differed between children with ASD with ID and those without. However, on the NP-MOT battery, neither of the two groups did well in the bi-manual and finger praxia tests (36 and 52% respectively failed). Manual and digital gnosopraxia showed some deficit (63 and 62% respectively failed). Postural deficits were found in tests for both static equilibrium (64%) and dynamic (52%). There were also difficulties in coordination between the upper and lower limbs in 58% of children. We found 75% failure in motor skills on the M-ABC test. Concerning muscular tone, significant laxity was observed in distal parts of the body (feet and hands), but hypertonia was observed in the proximal muscles of the lower limbs (reduced heel-ear angle).

**Discussion:** The results of manual and digital gnosopraxia tests point to a planning deficit in children with autism. A gesture programming deficit is also highlighted by the poor results in manual praxis, and by failures in the M-ABC tests despite prior training of the child. However, concerning global motor function, a significant difference was observed between children with and without ID. Our findings suggest a semiology of tone deregulation between proximal versus distal muscles, indeterminate tonic laterality, postural control deficit (proprioceptive), impairment of inter-hemispheric coordination (corpus callosum), and neurological soft signs such asdysdiadochokinesia, which leads us to hypothesize a general impairment of motor functions.

## Introduction

Autism Spectrum Disorders (ASD) are neurodevelopmental disorders that affect the child's development at an early stage, and persist in adulthood. There is today agreement on the fact that the origin of these disorders is multifactorial. They are characterized by pervasive impairments in several areas of development, and are today a major public health and societal issue on account of their considerable prevalence.

The diagnosis at present is based on pluri-disciplinary clinical evaluations, while early screening, tailored facilities, care provision and interventions rely on semiological knowledge of the disorders encountered in ASD.

Research has focused a good deal on cognitive features, social skills, and emotional aspects in children with ASD, while motor aspects have thus far received little attention. However, in recent years studies on the motor or sensory-motor behaviors of children with ASD have been undertaken, evidencing anomalies in motor function, in particular at a very early age.

Several studies have shown particular features in early motor development from the analysis of family videos, or from the assessment of motor development among children with suspected autism (Adrien et al., 1993; Maestro et al., 2005; Provost et al., 2007). Kanner in his initial description of autism in 1943 already mentioned signs of abnormal motor function in the first months of life, with babies exhibiting difficulties positioning their body when carried, and hypotonia.

Concerning the motor behaviors most frequently reported in ASD there are imitation disorders, static and dynamic postural balance difficulties, diminished postural control and compensation strategies to maintain balance (for a review of the literature see Paquet et al., 2015). Movement disorders are often downplayed in people with ASD, while their impact is significant in relationships with others and in other skills as adaptability to the environment or cognitive tasks. Many children with ASD have disorders in general dynamic coordination affecting locomotion, jumping, and dynamic balance. These types of coordination are essential in position changes (change from sitting to standing, compensating for its imbalance) and strength transfer (hitting a ball, lifting, moving an object). They include combinations of symmetrical or asymmetrical actions involving the left and right parts of the body, upper and lower limbs in association or dissociation. Coordination difficulties need to be defined from a detailed semiology in order to propose targeted therapeutic orientations. Several studies using the Movement Assessment Battery for Children (M-ABC) first or second edition (Henderson and Sugden, 1992; Henderson et al., 2007) reported overall difficulties in all three domains explored in this battery—manual dexterity, ball control, static, and dynamic balance—without however targeting the underlying brain function affected (Green et al., 2002, 2009; Hilton et al., 2007; Liu and Breslin, 2013). Considerable general impairment of motor functions was noted among subjects with ASD in an American meta-analysis (Fournier et al., 2010) and the authors concluded that coordination deficits can be considered as a major symptom in ASD. Finally, some authors suggest that Developmental Coordination Disorder (DCD) could also be a symptom of ASD (Dziuk et al., 2007; Dowell et al., 2009).

In several studies, only children with Asperger Syndrome or High functioning autism, or ASD children without intellectual disabilities, were included (Green et al., 2002; Jansiewicz et al., 2006; Dziuk et al., 2007; Freitag et al., 2007; Pan et al., 2009; Van Waelvelde et al., 2010; Whyatt and Craig, 2012; Liu and Breslin, 2013). However, other studies including ASD children irrespective of cognitive of status, have shown that motor disorders were more frequent in ASD children with poor cognitive skills (Mandelbaum et al., 2006; Green et al., 2009; Staples and Reid, 2010).

Thus, it appears that recent research aiming to further understanding of motor function among children with ASD reports global motor disturbances, but does not enable any understanding of the type and nature of the deficits. Indeed, most studies use motor skill evaluation tools such as the M-ABC battery, but these batteries are not developmental and assess global performances that are affected by learning, for example involving practice before the test. These batteries do not, on the other hand, enable symptom profiles to be highlighted, in particular minor deficits (neurological soft signs) that may be missed within the overall evaluation of general motor skills.

The neuro-developmental processes related to the maturing of the central nervous system have thus not been widely explored among children with ASD. Yet it is these processes that underpin motor performances, and could enable an understanding of the origin of the motor impairments encountered in these children. Thus, the semiology of the neuro-motor disturbances requires clarification, and the neurodevelopmental trajectory of neuro-psychomotor functions is not well mapped out in ASD children. In the present study we set out to perform a fine, discriminant analysis of each neuro-psychomotor and sensory-motor function so as to enable a better understanding of the nature of the psycho-motor disturbances in these children. To achieve a precise identification of the functions that are specifically affected, we used a standardized French developmental battery of the neuro-functional type, the NP-MOT (evaluation of neuro-psychomotor function in children; Vaivre-Douret, 2006). The other batteries or scales in French do not enable this type of discriminant analysis of motor and psychomotor function.

## Materials and methods

### Participants

Children with ASD aged 4–11 years, regardless of their level of intellectual ability, were recruited following consultations for the diagnosis of autism in the Autism Resource Center of Limousin, the Autism Resource Center of Aquitaine, and the University Center for Child and Adolescent Psychiatry of Limoges (France) between October 2013 and April 2015. All children complied with the criteria for the diagnosis of Pervasive Developmental Disorders according to DSM-TR-4 and the criteria of the Autism Diagnostic Interview-Revised (ADI-R; Lord et al., 1994), administered by a child psychiatrist specialized in ASD diagnosis. Thirty-four children were included in the study (31 boys and 3 girls, mean age 89 ± 28 months) among whom 18 with a diagnosis of Autism Disorder (AD; aged 81 ± 29 months), nine with a diagnosis of Pervasive Developmental Disorder not otherwise specified (PDD NOS; aged 106 ± 26 months) and seven with Asperger Syndrome (AS; aged 88 ± 22 months). Fifteen children had intellectual disabilities (44%; intelligence quotient IQ < 70), among whom 10 with AD and 5 with PDD NOS. Following the new classification proposed by the DSM-5 (APA, 2013) and after a new review of records, all children recruited met the criteria for ASD under the new nosographic classification. Children with motor disabilities of lesional or accidental origin, proven genetic disease or confirmed neurological disease were not included. The study was approved by an ethics committee (CPP-AC15-007).

To enable us to compare the performance of the 34 children with ASD with the performance of children with typical development, we used the results obtained by the children who formed the calibration group for the NP-MOT battery as reference values, (hereafter G Ref). The calibration group for the NP-MOT battery was composed of 446 French children, aged 48–101 months. All children were born full term, they had a height-weight development corresponding to the normal average by the standards of Sempé et al. (1979). All were schooled in classes corresponding to their age, without repetition or class jump. None of the children had motor or sensory disorders and no particular learning difficulties. The sample for calibration was divided into 5 age groups using the normal Gaussian distribution (group 1: 48–57 months inclusive; group 2: 58–68 months inclusive; group 3: 69–78 months inclusive; group 4: 79–89 months inclusive; group 5: 90–101 months inclusive) and for each group mean and standard deviation were calculated. Calibration tables were established for each test. The gender distribution was balanced in each age group and the results of the different evaluations did not evidence any significant influence of gender factor on any of the tests.

In this descriptive study, each child with ASD was compared to the reference standards according to their age group.

### Measures

The clinical data used in this study was derived from assessments conducted by a multidisciplinary team (child psychiatrist, neurologist, psychologist). Specific data for the neuro-psychomotor functions was derived from assessments of the children by a psychomotor therapist, using an evaluation of neuro-psychomotor function in children: The NP-MOT battery (Vaivre-Douret, 2006; Vaivre-Douret et al., 2011).

### Cognitive and psychomotor measures

The tests used to assess cognitive and psychomotor functions are presented in Table 1.

**Table 1:** **Cognitive and psychomotor measures**.

**Measures**	**Tests**
**COGNITIVE MEASURES**	
Cognitive functions	KABC-II or WISC-IV
Visuo-spatial-motor structuring	Rey's figure
Cognitive planning	Tower subtest of NEPSY
Visual perceptions and simultagnosis	Tangles figures
**PSYCHOMOTOR MEASURES**	
Gross motor skills	Movement-ABC
Manual and digital gnosopraxis (ideomotor praxis)	The gesture imitation: EMG

*KABC-II, Kaufman Assessment Battery for Children, Second Edition [KABC-II; (Kaufmann and Kaufmann, 2008)]; WISC-IV, Wechsler Intelligence Scale for Children, Fourth Edition [WISC-IV; (Wechsler, 2006)]; Movement-ABC, Movement Assessment Battery for Children (Henderson and Sugden, 1992); EMG, Evaluation de la Motricité Gnosopraxique (Vaivre-Douret, 1997)*.

Scores on the cognitive scales (KABC-II or WISC-IV) enabled classification of the children in two groups, those with intellectual deficiency and those without. Children with an IQ < 70 were classified in the group with intellectual deficiency (ID), those with an IQ > 70 were classified in the group without ID. All cognitive measures were administered by trained psychologists and psychomotor measures (Table 1) by a trained psychomotor therapist.

### Neuro-psychomotor measures

Neuro-psychomotor functions were assessed using the NP-MOT battery. This assessment and the above psychomotor assessment were administered by a trained psychomotor therapist. Demonstrations, verbal explanations, and/or picture were in some case used to help the children to understand the tasks required of them.

This battery enabled us to explore nine functions of neuro-psychomotor integration: Muscular tone, general motor skills, manual dexterity, laterality (tonic, spontaneous gestural, psychosocial, and usual), bodily spatial integration, manual praxis, tactile gnosis, rhythm, and auditory attention. It provides information on maturation levels for each of the functions explored, independently from other functions. The functions explored by the NP-MOT entail one or more tests, some of which consist of several items. Regarding specificity, the NP-MOT battery is a standardized normative instrument with identical subtests for any age (with expected saturation for subjects aged 8 years or more) to measure developmental maturation with qualitative assessments (movements) and quantitative assessments (speeds) for each item of the nine functions explored. Overall test-retest reliability of the NP-MOT has been reported to range from 70 to 98% and correlation coefficients with the LOMDS [similar to the Bruininks-Oseretsky Test of Motor Proficiency (Bruininks, 1978) for upper-limb coordination, balance and bilateral coordination subtests] were 0.72 and 0.84. The examination of muscular tone uses “dangling” (the degree of fluidity of movement of a body segment) and “extensibility” (the degree of mechanical stretching of a muscle and the opening of a joint angle). This enables evaluation of muscle resistance. The comparison of resistance by right/left body parts determines the more tonic side, referred to as dominant, and thus determines neurological tonic laterality. Gross motor-control tasks (dynamic and static) are scored for posture of the body and limbs and balance performances. A score of general limb coordination (between upper and lower limbs) is recorded for flying jump (i.e., jumping with feet together), spontaneous walk, and a score for postural control on landing after a jump (landing with two feet together). The exploration of laterality concerns three types—the laterality of spontaneous gestures, usual laterality, and psychosocial laterality. Footedness and dominant eye are also determined. The manual praxis examination contains different tasks. Bimanual tasks enable assessment of timing and ability to sequence the performance of repetitive and alternating movements, the ability to copy an action demonstrated by the examiner with bilateral hand pronation-supination, and symmetrical and asymmetrical movements, and the ability for digital praxis tasks, for instance speed of repetitive index-finger-thumb touching for each hand, and successive touching thumb to fingertips for each hand, eyes open. For the digital perception task (gnosis), the child is asked to show the localization of digital tactile stimuli on his concealed hand. The two hands are tested. Manual dexterity is assessed for each hand, recording time and fine motor quality. The task consists in putting a row of twelve counters into a box one by one as fast as possible. Body spatial integration (right and left) is assessed in relation to self by pointing (“show me your right arm”) and verbal command for axial crossing gesture (“put your right hand on your left ear”), and in relation to others, to objects and to a map or plan. Rhythmic tasks are performed first using the spontaneous rate of regular hand taps on the table. Then, an auditory-visual-kinesthetic adaptation task is required via imitation of patterns of hand or foot tapping by the examiner. The next task consists in auditory-perceptual-motor rhythmic adaptation. The child claps his hands, attempting to match the rate set by a metronome (3 speeds). Then, the child is asked to suit his walk to the same speeds. Auditory-attentional tasks consist in a series of 16 taps in a Go*/*No-Go task. The child taps with a stick only once when the examiner taps twice with a stick and twice when the examiner taps once (Vaivre-Douret, 2006 and Vaivre-Douret et al., 2011 for more details of the tests).

### Procedure

Only children for whom we received written consent from the parents and agreement from the children were included. Sixty-three percent of families (34 out of 54) agreed for their child to participate. The children were tested individually by the psychomotor therapist trained in the use of the NP-MOT battery. There was no order of execution for the different functions tested and the tests were independent from each other. The instructions were given verbally but visual material was provided with instructions for the tests for muscular tone and manual dexterity to facilitate understanding among children with comprehension difficulties. The tests were filmed in order to collect and analyse the data. Each test where the child obtained a result (whether failed or not) was described as achievable or administrable for the child. The results below 1 standard deviation were considered to be below the reference standard.

Data on early childhood development was derived from the children's medical records. The cognitive data were collected in the course of the consultation or during a follow-up assessment for diagnosis of ASD.

### Statistical analysis

Statistical analyses were conducted on SPSS® 21 (Statistical Package for Social Science) commercial software for Windows on PC. Categorical variables were presented as percentages and numbers, quantitative variables were presented as means and standard deviation. Comparisons of categorical variables were performed using Fisher's exact tests, and the means for quantitative variables using the *T*-test. For correlations, Spearman coefficients were used for paired items, because sample distribution did not follow a normal distribution. For each statistical analysis the significance threshold chosen was 5%.

## Results

### Description of the population

The characteristics of the children are presented in the table below (Table 2).

**Table 2:** **Characteristics of children with Autism Spectrum Disorder (ASD)**.

	**ASD (*m* ±*SD*)**
Age (months)	89 ± 28
Term (weeks' gestation)	39 ± 2
Sitting position (months)	8 ± 1
Age at first steps (months)	15 ± 7
Gender (M/F)	31/3
Medical history (%)	24
Problem *in utero* (%)	17
Psychomotor care (%)	70
Duration of psychomotor therapy follow-up (months)	17 ± 19
Intellectual deficiency (%)	44

The average age of the children was 7 years and 4 months. Five children were born prematurely (before 37 weeks' gestation) among whom one was born severely premature (32 weeks). All had an Apgar score of 10 at birth. Eight children (24%) had a medical history of mainly organic type (inguinal hernia, asthma, fracture, esophagitis). Four children presented fetal distress in *utero* or suspected fetal distress and one presented intrauterine growth retardation. The percentage of children with ASD who received psychomotor therapy because of corporeal or motor difficulties, and the duration of their treatment, evidences the overall psychomotor difficulties faced by the children in our sample. The average level of intellectual ability was not calculated because 11 children could not complete standardized cognitive assessments on account of intellectual deficiency. These 11 children were ranked among the 15 children (44%) having an intelligence quotient (IQ) < 70 (− 2 Standard Deviation). Sixteen children had an IQ between 70 and 130. Three had an IQ > 130 (+ 2 Standard Deviation).

### Descriptive analysis

Neuro-psychomotor examinations showed distal hypotonia of the upper limbs (wrist) (dangling and extensibility) (*p* < 0.001) and lower limbs (ankles) (dangling) (*p* < 0.001) characterized by absence of resistance between the two hemibodies or high angular values (compared with our reference, G Ref). Hypotonia was also found proximally for the upper limb (shoulder; *p* < 0.001). Axial physiological hypertonia was evidenced by poor curvature of the trunk (*p* = 0.002) but this was not observed among children with Asperger's syndrome and a restricted heel-to-ear angle (*p* < 0.001) were observed among children with ASD (Table 3). Tonic laterality of the upper limbs was significantly more indeterminate among ASD children than in our reference population (*p* < 0.001) but no difference was evidenced for tonic laterality of the lower limbs. Distal spasticity was observed in two children in the lower limbs and these same children had a restricted heel-to-ear angle and poor trunk curvature. The marked extensibility of the trunk observed in 18% of the children with ASD was similar to the G Ref observations. Screening for synkinesia using the diadochokinesia test evidenced adiadochokinesia in 57% of the children (*p* < 0.001) and marked synkinetic movements (co-movements and mirror movements) and oro-facial synkinesis.

**Table 3:** **Distribution of failure in the main tasks for neuro-psychomotor assessment and comparison with the reference standards for the NP-MOT battery (scores < 1 *SD*)**.

**Task**	**Proportion of failure by ASD children (*n*)**	**Proportion of failure by G Ref (*n*)**	***p*-value**
**Muscular tone**			
→ Hypotonia (distal upper limbs)	66% (22/33)	29% (131/446)	**<0.001**
→ Hypotonia (proximal upper limbs)	42% (13/31)	13% (57/446)	**<0.001**
→ Hypotonia (distal lower limbs)	97% (33/34)	55% (245/446)	**<0.001**
→ Hypertonia (proximal lower limbs)	9% (3/34)	0% (0/446)	**<0.001**
→ Hypotonia/Hypertonia (axial)	18% (6/34)/21% (7/34)	15% (68/446)/5% (21/446)	0.629/**0.002**
Motor pathway disorder (mild sign)	6% (2/34)	NS	
**Tone dominance**			
→ Indeterminate upper limb dominance	61% (19/31)	28% (126/446)	**<0.001**
→ Indeterminate lower limb dominance	39% (12/31)	26% (115/446)	0.140
Dysdiadochokinesis	57% (16/28)	26% (118/446)	**<0.001**
Synkinetic movements	36% (10/28)	73% (325/446)	**<0.001**
Oro-facial synkinesis	85% (23/28)	48% (216/446)	**<0.001**
**Manual dominance**			
→ Strong dominance	59% (16/27)	66% (294/446)	0.533
→ Weak dominance	37% (10/27)	33% (146/446)	0.675
→ Indeterminate dominance	4% (1/27)	1% (6/446)	0.339
→ Right handedness	70% (19/27)	94% (419/446)	**<0.001**
→ Left handedness	26% (7/27)	5% (21/446)	**<0.001**
Homogeneity between the upper and lower limbs	68% (19/27)	82% (371/446)	0.113
**Static balance**	64% (16/25)		
→ Feet joined	4% (1/26)	3% (12/446)	0.525
→ On one foot	63% (19/30)	36% (161/446)	**0.005**
→ On tip-toes	43% (12/28)	23% (103/446)	**0.023**
**Dynamic balance**	52% (14/27)		
→ Walk a line forward/backward	60% (18/30)/79% (22/28)	25% (114/446)/33% (149/446)	**<0.001/<0.001**
→ Walk on tiptoe	16% (2/32)	13% (59/446)	0.408
→ Jump: coordination/landing	50% (17/34)/53% (18/34)	11% (47/446)/13% (57/446)	**<0.001/<0.001**
**Manual dexterity**	33% (10/30)	17% (74/446)	**0.027**
**Manual praxis**	36% (8/22)		
→ Bi-manual praxis symmetric and asymmetric; quality/duration	54% (14/26)/34% (9/26)	52% (232/446)/0% (0/446)	1/ **<0.001**
→ Thumb/index opposition: right/left	4% (1/24)/44% (10/23)	2% (10/446)/2% (10/446)	**<0.001/<0.001**
→ Thumb/finger opposition: right/left	26% (6/23)/26% (6/23)	2% (10/446)/2% (10/446)	**<0.001/<0.001**
**Bodily spatial integration**			
→ Bodily spatial integration to self: designation/order with crossing of the body midline	28% (8/29)/50% (14/28)	15% (67/446)/20% (79/392)	0.108/ **<0.001**
→ Bodily spatial integration to other: designation/imitation with crossing of the body midline	55% (16/29)/52% (12/23)	43% (194/433)/21% (58/274)	0.336/**0.002**
→ Bodily spatial integration to object: 2 objects/3 objects	27% (6/22)/68% (15/22)	26% (71/274)/34% (93/274)	1/**0.002**
**Rhythm task**			
→ Auditory-perceptual-motor task: taping in rhythm/walking in rhythm	23% (6/26)/36% (9/25)	31% (138/446)/41% (182/446)	0.513/0.681
→ Auditory-visual-kinesthetic task	38% (10/26)	18% (79/446)	**0.017**
→ Tapping	14% (4/28)	33% (147/446)	0.057
**Digital perception**	9% (2/23)	11% (48/446)	1
**Auditory attention:** time/quality	45% (9/20)/20% (4/20)	0% (0/446)/0% (0/446)	**<0.001/<0.001**

*NS, not specified; Bold values indicate significant p-values*.

Concerning hand preference, there was a significant difference (*p* < 0.001) between the ASD sample and G Ref, with a proportion of left-handed ASD children in G ASD (26%, *n* = 7) larger than in G Ref (5%, *n* = 21; for detailed results of these comparisons see Paquet et al., submitted). Static equilibrium was impaired in 64% of the children in our sample. The postural control task with the two feet together and eyes closed did not differentiate ASD children from G Ref. Balancing on one leg with eyes open evidenced lesser balance control on one foot for children with ASD (*p* = 0.005). The time maintaining postural balance (qualitative aspects) was significantly shorter among ASD children for all tasks (balancing with feet together, on one foot, on tiptoe). Dynamic equilibrium, a test failed by 52% of the ASD children, evidenced postural difficulties, difficulties in speed of execution in, walking heel-toe on a line and walking on the heels, where the extension of the arms was characteristic among our ASD children. Walking on tiptoe was well-performed. In normal walking, the “toe-walking” characteristic of autism was observed in 7 children. The jump test was mainly failed by our ASD children, characterized by an asymmetric jump (*p* < 0.001), absence of coordination between upper and lower limbs (*p* < 0.001) and postural imbalance on landing (*p* < 0.001). For the manual dexterity test children with ASD had more praxis difficulties in gripping the token than the G Ref. The manual praxis tests, which were not performed by all the children, showed better performances in the thumb-index opposition test in comparison with bi-manual praxis (symmetrical and asymmetrical) and the thumb-finger opposition task. From a quantitative viewpoint, for each test, children with ASD were slower in their execution of the movement than the G Ref. The performances on the symmetrical bi-manual pronation-supination tests were less well synchronized among children with ASD, and when they were synchronized, the movement tended to be of proximal origin. Overall failures in opposition between thumb and the other digits were more marked among children with ASD, for both left and right hand. The rate of failure in the gesture imitation test for hands and digits (manual and digital gnosopraxis) was high (respectively 63 and 62%; Table 4). Bodily spatial integration tests showed poorer left-right integration for ASD children (compared with G Ref), with significant failure in the verbal command task for the axial crossing gesture (*p* < 0.001). The imitation test involving reversibility was also less well performed by ASD children (52% failure, *p* = 0.002). No differences were evidenced between ASD children and G Ref for spatial integration when two objects were presented, but differences appeared with three objects. The children with ASD failed more often than the G Ref children. Orientation from a plan did not show any differences. The test for matching a kinesthetic/visual/auditory rhythm was more often failed among ASD children than the G Ref. In this test, the ASD children failed more often on rhythms involving inter-segment dissociation between the two hemibodies (*p* = 0.017). The sustained auditory attention test was significantly more often failed by ASD children (*p* < 0.001).

**Table 4:** **Distribution of failure in the main tasks for gross motor and neuropsychological assessment**.

**Task (battery)**	**Proportion of failure by ASD children (*n*)**	
	****	
	**< 2 SD**	**< 1 SD**
**GNOSOPRAXIS (EMG)**		
→ Manual	53% (16/30)	10% (3/30)
→ Digital	41% (12/29)	21% (6/29)
**GROSS MOTOR SKILLS: total (M-ABC)**	**41% (12/28)**	**32% (9/28)**
→ Manual Dexterity	32% (9/28)	21% (6/28)
→ Ball skills	20% (6/30)	30% (9/30)
→ Static and dynamic balance	34% (10/29)	24% (7/29)
**VISUO-MOTOR COORDINATION (NEPSY)**	**19% (6/31)**	**19% (6/31)**
**VISUAL PERCEPTION**		
Simultagnosis	11% (3/27)	7% (2/27)
**NEUROPSYCHOLOGICAL FUNCTIONS**		
Visuo-spatial-motor structuration (Rey's figure)	23% (6/26)	27% (7/26)
Cognitive planning (NEPSY, Tower subtest)	26% (6/23)	22% (5/23)

Results for gross motor skills (M-ABC; Table 4) were also poorer among ASD children in each of the three areas explored by this battery. Visual perception was little affected in the sample. Cognitive tests for visuo-spatial motor structuring and cognitive planning were frequently failed (Table 4).

Significant differences were observed depending on presence or absence of ID among ASD children. ASD children with ID obtained significantly poorer results for the following tests: Static balance (time), Dynamic balance (posture and coordination), Hand-eye skill (execution speed), Thumb/finger opposition, Manual gnosopraxis, Bodily spatial integration (all tasks), Rhythm task, Gross motor skills (M-ABC), Visuo-spatial-motor structuring, and Cognitive planning (Table 5).

**Table 5:** **Distribution of failure in the main tasks for neuro-psychomotor, gross motor, and neuropsychological assessment according to the presence of intellectual deficit (ID; ns, non-significant)**.

**Task (battery)**	**Proportion of failure by ASD children with ID (*n*)**	**Proportion of failure by ASD children without ID (*n*)**	***p*-value**
**NEURO-PSYCHOMOTOR FUNCTIONS**			
**Muscular tone (NP-MOT)**			
→ Hypotonia (distal upper limbs)	72% (10/14)	62% (12/19)	0.719
→ Hypotonia (proximal upper limbs)	46% (6/13)	39% (7/18)	0.727
→ Hypotonia (distal lower limbs)	23% (3/14)	26% (5/19)	1
→ Hypertonia (proximal lower limbs)	47% (7/15)	47% (9/19)	1
→ Hypotonia/Hypertonia (axial)	13% (2/15)/27% (4/15)	21% (4/19)/16% (3/19)	0.672/0.672
Motor pathway disorder (mild sign)	13% (2/15)	0% (0/19)	0.187
Dysdiadochokinesis (NP-MOT)	80% (2/10)	50% (9/18)	0.226
Synkinetic movements (NP-MOT)	40% (4/10)	33% (6/18)	1
**Manual dominance (NP-MOT)**			
→ Strong dominance (NP-MOT)	56% (5/9)	61% (11/18)	1
→ Weak dominance (NP-MOT)	33% (3/9)	39% (7/18)	1
→ Indeterminate dominance (NP-MOT)	11% (1/9)	0% (0/18)	0.333
→ Right handedness (NP-MOT)	67% (6/9)	72% (13/18)	1
→ Left handedness (NP-MOT)	22% (2/9)	28% (5/18)	1
**Static balance (NP-MOT)**	100% (7/7)	50% (9/18)	**0.027**
**Dynamic balance (NP-MOT)**	89% (8/9)	33% (6/18)	**0.013**
→ Walk	89% (8/9)	33% (6/18)	**0.013**
→ Jump	87% (13/15)	42% (8/19)	**0.013**
Upper limb/lower limb coordination (NP-MOT)	85% (11/13)	39% (7/18)	**0.025**
**Hand-eye skill (NP-MOT)**	45% (5/11)	10% (2/19)	0.068
**Manual praxis (NP-MOT)**			
→ Bi-manual praxis symmetric and asymmetric	71% (5/7)	22% (4/18)	0.058
→ Thumb/index opposition	12% (1/8)	19% (3/16)	1
→ Thumb/finger opposition	87% (7/8)	33% (5/15)	**0.027**
Manual gnosopraxis (EMG)	92% (11/12)	44% (8/18)	**0.018**
Digital gnosopraxis (EMG)	82% (2/11)	50% (9/18)	0.125
**Bodily spatial integration (NP-MOT)**			
→ Bodily spatial integration to self	91% (10/11)	17% (3/18)	<**0.001**
→ Bodily spatial integration to other	89% (8/9)	0% (0/15)	<**0.001**
→ Bodily spatial integration to object	71% (5/7)	7% (1/15)	0.004
**Rhythm task (NP-MOT)**			
→ Rhythm task (auditory-perceptual-motor task)	25% (2/8)	18% (3/17)	1
→ Rhythm task (auditory-visual-kinesthetic task)	89% (8/9)	23% (4/17)	**0.003**
→ Tapping	30% (3/10)	5% (1/18)	0.116
**Digital perception (gnosis NP-MOT)**	17% (1/6)	0% (0/17)	0.261
**Auditory attention (NP-MOT)**	75% (3/4)	25% (4/16)	0.101
**Gross motor Skills**			
Total gross motor skills (M-ABC)	61% (11/18)	100 (10/10)	**0.030**
→ Manual dexterity	44% (8/18)	70% (7/10)	0.254
→ Ball skills	28% (5/18)	83% (10/12)	**0.008**
→ Static and dynamic balance	50% (9/18)	73% (8/11)	0.273
**Visual Perception**			
Simultagnosis	0% (0/17)	33% (4/11)	**0.016**
**NEUROPSYCHOLOGICAL FUNCTIONS**			
Visuo-spatial-motor structuration (Rey's figure)	28% (5/18)	100 (8/8)	**0.002**
Cognitive planning (NEPSY, Tower)	33% (7/19)	100% (5/5)	**0.037**

*Bold values indicate significant p-values*.

## Discussion

This study has made it possible to show the poor results in neuro-psychomotor functions and gross motor skills among ASD children. The results obtained on the NP-MOT concerning tonus are particularly interesting. We found no data in the literature on the specific evaluation of tone in children with ASD, and when tone is broached in studies on motor function, the method for exploring muscle tone is often vague and lacking in detail, with no reference norms for children. Only a few studies, involving a neurological examination or investigating walking in people with autism, have reported data on muscle tone (Shetreat-Klein et al., 2014). Studies on general aspects of motor function only rarely report data on tone in children with ASD.

In resting tone we noted high angle values for shoulder and wrist, in line with the results of Shetreat-Klein et al. (2014) who in a recent study compared the joint angles of wrists, fingers, and ankles in 38 children with ASD, comparing them with a control group in the absence of norms for tone in children in the USA. Hypotonia was also described by Ming et al. (2007) but the way in which resting tone was assessed is not specified in their study.

For the lower limbs we noted greater distal ligament laxity, but hypertonia was evidenced proximally, involving the buttocks and hamstring muscles. These muscles are involved in stabilization of the pelvis. Hypertonia was also noted in the trunk. Our observations on muscle tone could suggest the existence of motor abnormalities in the ventral cortico-spinal tract from the cerebral cortex or the median pyramidal tracts from the brainstem, these being responsible for the adjustment of proximal muscles and also posture.

As seen above, 64% of the children assessed exhibited poorer static balance compared to the reference values. Several studies based on clinical evaluations using standardized tools report similar results. Difficulties maintaining balance for any length of time and compensation with the arms, evidencing postural immaturity, point to a global deficit in posture control. The coordination of dynamic balance also shows disturbances for more than half the children (52%), in particular failure to stabilize on landing from a jump, again showing disturbances in postural adjustment. On the M-ABC battery, we also noted marked overall failure for static and dynamic balance.

The observations from the NP-MOT battery lead us to hypothesize a delay or a disturbance in maturation of postural control under the influence of the basal nuclei and the cerebellum. It would be interesting to explore this hypothesis using neuroimagery investigation in combination with a standardized assessment tool for neuro-psychomotor functions. These postural difficulties seem to us to be more likely to relate to a disharmony in the adjustment and regulation of tone mentioned above, which could lead to disturbances in proprioceptive sensitivity, rather than malfunction of vestibular origin.

Our results concerning coordination and manual praxis show good performances in fine coordination (manual dexterity) but bi-manual and praxis difficulties in the pronation-supination bimanual tests and in opposition between thumb and the other fingers, evidencing difficulties in motor planning. We also observed difficulties in more than 60% of the sample in the gesture imitation task (manual and digital gnosopraxis) also suggesting motor planning difficulties. We noted variable results for the other praxis tests, in line with the observations by Miller et al. (2014), and praxis difficulties linked to general motor function impairments.

Digital tactile gnosia, perturbed in DCD (Vaivre-Douret et al., 2011) was not impaired in our ASD sample.

Although the results for global motor function were also variable, and dependent on presence or absence of ID, the majority of the ASD children exhibited poor regulation of muscle tone and poor postural control linked to proprioception, along with tonic disharmony, and problems of inter-hemispheric motor coordination involving the corpus callosum. Thus, there seems to be a general disturbance in functions affecting gross motor coordination, bi-manual coordination, accompanied by minor neurological dysfunction/neurological soft signs such as dysdiadochokinesia.

Our results suggest a general disturbance in coordination affecting different brain function networks.

We also observed rates of failure on visuo-spatial tests that were strongly linked to the presence of ID, which could suggest a dysexecutive disorder, given the failures on Rey's figure and the Tower test for cognitive planning among children with ID in particular.

### Neuro-psycho-physiological hypotheses for the psychomotor and neuro-psychomotor features described

As we have seen, there is dysfunction of postural adjustments and tonic organization in most of the children in the cohort, pointing to a possible implication of the cerebellum acting as a motor-control organ. We hypothesize, as other authors have suggested for DCD, subcortical implication with repercussions on the cortical structures (Vaivre-Douret et al., 2011; Zwicker et al., 2012). However, although structural and functional anomalies of the cerebellum have been described in subjects with ASD (Allen et al., 2004; Wegiel et al., 2014), which could partly explain the dysfunctions observed, the implication of a single structure is fairly improbable. From the quantitative analysis of movements we noted difficulties in planning, anticipation, control and bilateral coordination of gestures, along with slowness, suggesting the possible implication of a wide network of cortical and sub-cortical structures, which does not fit with a specific DCD disorder (Lalanne et al., 2012).

The hypothesis of dysfunction in the thalamic nuclei could also be put forward, since this structure is at the crossroads of sub-cortical afferences and efferences toward the pre-motor associative zones, and also because of their integrating role for sensory pathways.

Finally, in our study, laterality, which can be viewed as the behavioral result of cerebral dominance, was affected, in particular laterality under the influence of hemispheric organization (26% left-handedness, 61% indeterminate upper limb tone dominance). Some studies (Kashiwagi et al., 2009; Króliczak and Frey, 2009) have evidenced the implication of the left hemisphere in praxis tasks. Another possible hypothesis is an anomaly in hemispheric lateralization, with delayed or disordered organization, which could explain some of the coordination or praxis disturbances encountered among these children, and which could also explain other disorders such as language disturbances observed in ASD.

The hypothesis of an anomaly in inter-hemispheric connectivity can also be put forward, and could explain the difficulties noted in bi-manual and rhythm tasks.

These different hypotheses open the way toward further research combining brain imagery and clinical observation, in order to look for correlations between the semiological characteristics of the motor disturbances observed and possible anatomical or functional cerebral anomalies.

### Profile

We found characteristics in common for a large number of children in this ASD sample. There does indeed appear to be an underpinning of disturbed tone in these children, whether or not they exhibit intellectual deficit, in particular on proximal-distal level, where deregulation of tone was observed in the majority of the children, and disturbed laterality linked to mostly indeterminate hemispheric organization. This common substrate of disharmony could affect all motor and psychomotor functions, particularly muscular tone which is the foundation on which movement emerges. But another factor may also be involved in the psychomotor disorders, that is to say ID, which could lead to differences in the degree of disturbance, or to more specific disturbances such as executive function disorders.

Given the large number of psychomotor difficulties among ASD children, establishing a psychomotor semiology seems just as important as knowledge of their cognitive profiles for an understanding of the functions affected, so as to propose therapeutic orientations that are better targeted.

### Limitations

This work has certain limitations, among which the small number in the sample in view of the numerous variables explored, and the occurrence of missing data as a result of intellectual deficit for some children, so that some tests could not be administered. Another limitation is the absence of a control group in this study.

### Perspectives

This study has enabled the description of a certain number of impaired neuro-psychomotor functions in a majority of children with ASD, in particular basic functions such as muscle tone or postural control. It seems to us that in the future it would be worthwhile exploring the impact of these basic functions on automatic and voluntary motor function, which would enable us to test the hypothesis of a sub-cortical influence on cortical structures.

In addition, our results concerning posture lead us to the hypothesis of a link between tonic disharmony and difficulties in controlling and adapting posture. It would nevertheless be interesting to also envisage the implication of the function of anticipation, which has been described by some authors as being disturbed in ASD.

A recent review of the literature has focused on the computational approach in motor control and suggests motor planning difficulties but also variability in sensory inputs and motor outputs (Gowen and Hamilton, 2013). Sensory aspects and possible sensory atypicalities were not investigated in our study on the basic neuro-psychomotor functions, but these seem to us important to explore in futures studies on specific motor skills.

## Conclusion

This study has enabled a common core of tonic deregulation and delayed or disturbed lateralization to be evidenced, alongside considerable disturbances in postural control and difficulties in tonic and postural adjustment in dynamic balance coordination. The analysis of fine coordination and praxis leads us to the hypothesis of a global disturbance in motor function, possibly linked to dysfunction of the sub-cortical structures. The common semiological basis evidenced for disharmony in muscular tone appears as an interesting phenotype indication which could provide an early developmental marker in these children, thus opening the way to earlier diagnosis.

It is the standardized neuro-developmental evaluation used here that enabled this semiology to be evidenced, providing a better understanding of the nature of the motor and psychomotor disturbances occurring, and enabling hypotheses as to the mechanisms involved. Using this improved knowledge of the nature of the disorders, new lines of research seem worth pursuing, in association with clinical observation, so as to cross perspectives with the different disciplines.

## Author contributions

AP conceived and designed the study, she has contributed to collect and analysis and interpretation of the data, contributed to draft the work, reviewed and approved the final version, and its submission. Her agreement has been accountable for all aspects of the work in ensuring that questions related to the accuracy or integrity of any part of the work are appropriately investigated and resolved. BO has substantial contributed to statistical analysis and interpretation of the data, and approved the final version to be published, and his agreement has been accountable for all aspects of the work. MB has substantial contributed to the design of the study, interpretation of the data, and to draft the work. He approved the final version to be published and his agreement has been accountable for all aspects of the work. BG participated to the conception of the study and to draft the work, approved the final version to be published, and his agreement has been accountable for all aspects of the work. LV participated to the design study, statistical analysis and interpretation of the data, and to draft the work, reviewed and approved the final version to be published, and her agreement has been accountable.

## Funding

This study was supported through funding provided by the Caisse primaire d'assurance maladie de la Haute-Vienne; the Fondation pour la recherche en psychomotricité et maladies de civilization.

### Conflict of interest statement

The authors declare that the research was conducted in the absence of any commercial or financial relationships that could be construed as a potential conflict of interest.
